# Detection method for munage grape clusters and abnormal berries under color-similar backgrounds

**DOI:** 10.3389/fpls.2026.1931877

**Published:** 2026-09-09

**Authors:** Xinzhao Zhou, Peisheng Wang, Haiyan Liu, Hong Jiang, Wen Zhang, Xiuzhi Luo, Xiaojuan Li, Xiangjun Zou, Jinlong Lin

**Affiliations:** 1 School of Mechanical Engineering, Xinjiang University, Xinjiang, China; 2 Forestry Administrative Station of Kirgiz Autonomous Prefecture, Artux, Xinjiang, China; 3 Changji Vocational and Technical College, Changji, Xinjiang, China; 4 Xinjiang Research Institute of Agriculture in Arid Areas, Urumqi, Xinjiang, China

**Keywords:** abnormal berry detection, color-similar background, local abnormal feature enhancement, munage grape, semantic-guided recall compensation, YOLO11n

## Abstract

Accurate detection of Munage grape clusters and abnormal berries in field scenes is hindered by several challenges: mature berries often exhibit colors similar to those of branches and leaves; cluster-level large targets coexist with medium- and small-scale abnormal berry targets; local abnormal cues within full-berry bounding boxes are easily diluted by responses from normal berry skin and waxy bloom; and shallow-level textures, specular highlights, and adjacent berry boundaries may induce false detections. To address these challenges, this study proposes YEIS, a YOLO11n-based detection method for Munage grape clusters and abnormal berries under color-similar backgrounds. Built upon YOLO11n, the proposed method first introduces EMBSFPN to construct multi-scale candidate features, thereby alleviating the scale-representation discrepancy between cluster-level targets and berry-level abnormal targets. Second, an intra-berry frequency–local evidence decoupling module, IB-FLED, is designed to enhance local abnormal cues within full-berry detection boxes through low-frequency appearance estimation, local residual modeling, and morphology-aware response branches. Finally, a semantic-guided recall compensation module, SGRCM, is developed to constrain P3 detail compensation using P4 semantic information refined by IB-FLED, reducing the interference of shallow-level textures, waxy bloom, and specular highlights in abnormal berry localization. Three random-seed experiments were conducted on a self-built field dataset. The results show that YEIS achieves Precision, Recall, mAP_50_, mAP_75_, and mAP_50–95_ values of 83.21%, 79.97%, 88.34%, 83.27%, and 76.91%, respectively. Compared with YOLO11n, the overall mAP_50–95_ is improved by 1.71 percentage points, and the mAP_50–95_ for lesion-like abnormal berries is increased by 1.61 percentage points. For scar-like abnormal berries, the F1-score and mAP_50–95_ are improved by 3.01 and 3.41 percentage points, respectively. Meanwhile, the number of model parameters is reduced from 2.583 M to 2.149 M, corresponding to a reduction of 16.8%. The proposed method improves the detection and localization of abnormal berries under color-similar backgrounds while reducing the parameter count relative to YOLO11n, providing a front-end visual detection approach for digital monitoring, grape-cluster localization, and abnormal-berry recognition in Munage vineyards.

## 1 Introduction

Munage grape is a representative local table-grape cultivar in Xinjiang, China, and is mainly distributed around Artux, Xinjiang (Zhong et al., 2021, 2026). This cultivar has high practical value in local table-grape production. With advances in artificial intelligence, computer vision-based fruit recognition and abnormality detection have become increasingly important for precision viticulture (Tardaguila et al., 2021; Mohimont et al., 2022). For Munage grapes, mature berries often have colors similar to those of surrounding branches and leaves, while the waxy bloom on their surfaces further reduces visual contrast. Lesion-like and scar-like abnormal berries usually account for only a small proportion of a cluster and often exhibit weak or indistinct boundaries. These characteristics make abnormal berries easy to overlook during manual inspection and can also lead to missed detections and false positives in general-purpose detectors. The detection difficulty is further increased by unstructured backgrounds, branch and leaf occlusion, illumination variation, dynamic interference, and densely distributed targets in natural field environments, all of which can reduce the stability of object recognition and localization (Tang et al., 2020, 2023). Therefore, a visual method capable of simultaneously detecting grape clusters and abnormal berries is needed to support vineyard monitoring, cluster localization, and field abnormality warning.

Traditional vision methods based on hand-crafted color, morphological, textural, and geometric features have provided a valuable foundation for grape and other fruit detection and measurement (Nuske et al., 2011; Font et al., 2015; Luo et al., 2016; Lin et al., 2020; Luo et al., 2021). Nevertheless, their performance may be affected by illumination variation, occlusion, low contrast, and color-similar backgrounds. With advances in deep learning, grape-related vision studies, particularly those based on one-stage detectors such as YOLO, have mainly investigated grape-cluster localization, counting, instance segmentation, and tracking (Santos et al., 2020; Lu et al., 2022b; Sozzi et al., 2022a, 2022b), as well as the localization of rachises, picking points, and other structural components for harvesting operations (Zhang et al., 2023; Chen J. et al., 2024; Zhou et al., 2025). However, relatively few studies have developed task-specific architectures for the simultaneous bounding-box detection of large grape-cluster targets and small abnormal-berry targets in color-similar backgrounds.

In the recognition of grape diseases and abnormal appearances, previous studies have explored classification (Liu et al., 2020; Tang et al., 2020), detection (Xie et al., 2020), and instance segmentation (Rossi et al., 2022). Other studies have focused on grape-cluster status assessment, biophysical damage evaluation, and pre-thinning berry counting (Du and Liu, 2023; Pinheiro et al., 2023). Nevertheless, most existing studies still target leaf diseases, cluster status, or disease discrimination at the whole-object level. For abnormal berries, existing approaches have more commonly employed reconstruction-based anomaly detection, autoencoders, or synthetic anomaly generation strategies (Strothmann et al., 2019; Miranda et al., 2022; Motoi et al., 2025), focusing on anomaly recognition, anomaly distribution modeling, or abnormal sample augmentation rather than joint bounding-box detection of grape clusters and abnormal berries in the same field image.

In agricultural object detection, improved feature representation is often accompanied by increased model complexity, which may restrict practical use (Li et al., 2021; Zhang et al., 2021). Attention mechanisms and efficient feature extractors have therefore been used to improve fruit detection in complex orchard environments (Lu et al., 2022a; Tian C. et al., 2025). However, indiscriminate cross-scale aggregation may amplify responses from waxy bloom, specular highlights, shadows, and adjacent berry boundaries. Cross-level semantic guidance is therefore needed to strengthen abnormal cues while limiting irrelevant responses.

Accordingly, this study proposes YEIS, a YOLO11n-based detector for Munage grapes under color-similar field backgrounds. The network incorporates an efficient multi-branch and scale feature pyramid network (EMBSFPN) (Gao et al., 2025) in the neck to enhance multi-scale feature fusion between grape clusters and abnormal berries. Motivated by the localized, low-contrast, and weak-boundary characteristics of lesion-like and scar-like abnormal berries, an intra-berry frequency–local evidence decoupling module (IB-FLED) is designed to strengthen local abnormal cues that are submerged by normal appearance responses within full-berry detection boxes. Furthermore, a semantic-guided recall compensation module (SGRCM) is developed to use IB-FLED-refined mid-level semantic information to guide shallow-level detail compensation, thereby improving candidate detection and localization stability for weak-boundary abnormal berries. The main contributions of this study are as follows:

1. A detection dataset for Munage grape clusters and abnormal berries is constructed from real orchard scenes with color-similar backgrounds. Grape clusters, lesion-like abnormal berries, and scar-like abnormal berries are annotated at the bounding-box level, providing a data basis for this task. The two types of abnormal berries are defined as visual labels according to visible abnormal appearances in images and are not used for pathological diagnosis or causal determination of damage.
2. A YOLO11n-based network, YEIS, is developed for simultaneous bounding-box detection of grape clusters and berry-level abnormal targets. IB-FLED and SGRCM address local abnormal-cue dilution and missed detection of weak-boundary berries, respectively, while the final model reduces the parameter count relative to YOLO11n.
3. Multiple random-seed experiments, main-module ablation studies, and internal mechanism ablations are conducted to evaluate the contribution of each module. The results show that YEIS improves the detection and localization of abnormal berries under color-similar backgrounds, particularly weak-boundary scar-like berries, while using fewer parameters than YOLO11n.

The remainder of this paper is organized as follows. Section 2 introduces the dataset construction, detection task characteristics, and YEIS network architecture. Section 3 presents the experimental analysis, including ablation experiments, detection results on complex field samples, error analysis, and comparison with representative models. Section 4 discusses the advantages, mechanisms, application boundaries, and future improvement directions of the proposed model. Section 5 presents the conclusions.

## 2 Materials and methods

### 2.1 Dataset and annotation protocol

Images of Munage grapes were collected from vineyards in Artux, Xinjiang Uygur Autonomous Region, China, from July to September 2025. RGB imaging devices, including a Huawei Mate 40 Pro and a Redmi K60, were used for image acquisition. After screening, size organization, and annotation, a detection dataset was obtained. The processed images were mainly 960 × 1280 and 720 × 1280 pixels. The dataset contains 1,878 images. The dataset was divided at the image level into training, validation, and test subsets at an 8:1:1 ratio. During partitioning, the class-instance distribution across GCLUSTER, major, and minor was kept approximately consistent among the three subsets, as summarized in **Table 1**. Each annotated image, together with all bounding boxes contained in that image, was assigned exclusively to one subset.

**Table 1:** Dataset split and class distribution for Munage grape clusters and abnormal berries.

Split	Images	Boxes	GCLUSTER	Major	Minor	Average targets/image
Training set	1504	15279	5322	5993	3964	10.16
Validation set	187	1945	676	764	505	10.40
Test set	187	1940	671	769	500	10.37
Total	1878	19164	6669	7526	4969	10.20

Three detection classes were defined in the dataset: GCLUSTER, major, and minor. GCLUSTER denotes a Munage grape cluster and is treated as a cluster-level target. The major class denotes lesion-like abnormal berries, whereas the minor class denotes scar-like abnormal berries; both are berry-level abnormal targets. All objects were annotated using rectangular bounding boxes, and the annotation files were saved in YOLO format.

**Figure 1:**
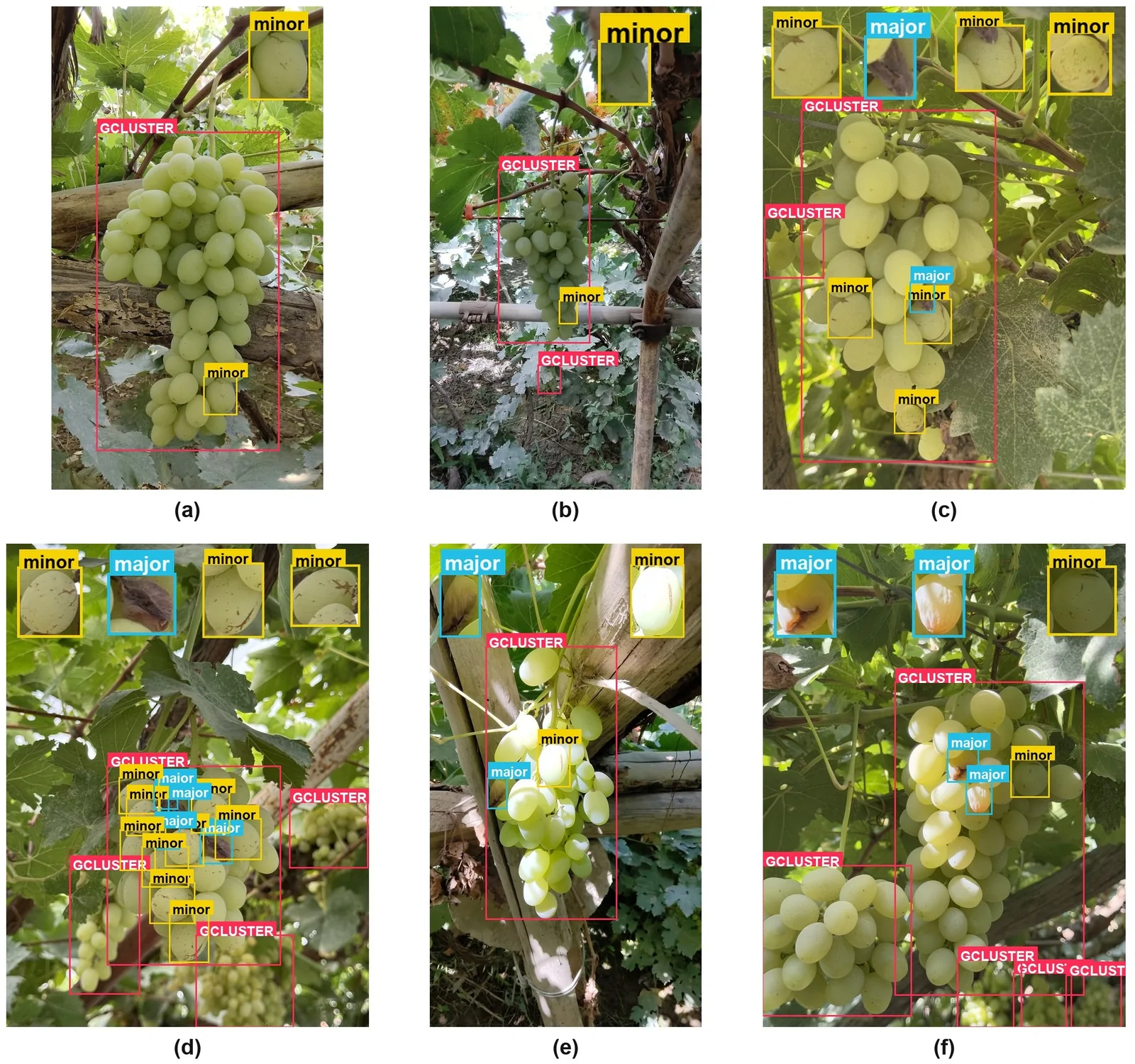
Typical challenging field samples and bounding-box annotation examples of Munage grapes. **(a)** sunny conditions; **(b)** overcast conditions; **(c)** clusters partially occluded by leaves; **(d)** clusters at different distances; **(e)** berries with surface highlights; and (f) adjacent clusters.

As shown in **Figure 1**, the dataset contains three bounding-box classes: GCLUSTER, major, and minor. GCLUSTER denotes grape clusters, whereas major and minor denote lesion-like and scar-like abnormal berries, respectively. The labels major and minor are defined solely according to visible appearance and do not represent pathological diagnoses, causal determinations, or quantitative severity grades.All three classes can coexist within the same image. Because abnormal berries are generally located within grape clusters, major or minor bounding boxes may spatially overlap with GCLUSTER bounding boxes. Such overlapping instances were retained as independent annotations without deletion or merging.

For GCLUSTER, the bounding box enclosed the main visible region of the cluster. GCLUSTER instances affected by branch or leaf occlusion, berry overlap, or background interference were retained when their main contours remained identifiable, whereas severely blurred instances or those with indistinguishable contours were excluded. For both major and minor, the visible region of the abnormal berry was used as the detection unit, while local lesion-like or scar-like regions were not annotated at the pixel level. Partially occluded abnormal berries were retained when their category remained recognizable, whereas instances with severe blur, uncertain boundaries, or ambiguous categories were excluded. When the same berry simultaneously exhibited lesion-like and scar-like abnormal appearances, it was annotated as major.

**Figure 1** presents typical challenging samples, including color-similar backgrounds, distant small targets, the coexistence of lesion-like and scar-like abnormalities, densely distributed abnormal berries, specular highlights, and overlapping grape clusters. In the figure, red boxes indicate GCLUSTER, blue boxes indicate major, and yellow boxes indicate minor, illustrating the typical visual interference and annotation format in the dataset.

To analyze scale differences among target classes, bounding-box sizes for each category were counted at an input size of 1280 × 1280. The results are shown in **Table 2**.

**Table 2:** Bounding-box scale statistics of different target classes at an input size of 1280 × 1280.

Class	Instances	Median size/pixel	Mean size/pixel	Median area	Small/%	Medium/%	Large/%	Short side< 64/%
GCLUSTER	6669	191.2 × 297.5	228.6 × 372.8	55766.2	0.04	11.23	88.72	7.18
major	7526	52.0 × 61.1	55.1 × 63.6	3150.8	8.58	85.64	5.78	73.19
minor	4969	70.2 × 75.7	73.0 × 78.0	5310.9	1.11	81.69	17.21	40.69

The Small, Medium, and Large categories in **Table 2** follow the scale definitions commonly used in COCO object detection evaluation (Lin et al., 2014). According to the bounding-box area *A*, targets with *A*< 32^2^, 32^2^ ≤ *A*< 96^2^, and *A* ≥ 96^2^ correspond to Small, Medium, and Large, respectively, where *A* is calculated under the 1280 × 1280 input scale. This scale analysis was used only to describe dataset characteristics and was not involved in training. As shown in the table, GCLUSTER is dominated by large-scale targets, with Large accounting for 88.72%. By contrast, major and minor are concentrated at small and medium scales. In particular, 73.19% of major instances have a short side smaller than 64 pixels, and the corresponding proportion for minor is also 40.69%. This distribution indicates that the task requires a single detection model to handle both cluster-level large targets and berry-level small- to medium-scale abnormal targets.

### 2.2 Detection task characteristics and structural design rationale

Detection of Munage grape clusters and abnormal berries first involves the coexistence of cross-scale hierarchical targets. GCLUSTER generally has a large spatial span and requires the model to preserve the overall cluster contour and contextual information. In contrast, major and minor are mostly located inside grape clusters, are smaller in size, and are more sensitive to local position and detailed features. Therefore, the model must retain structural representation capability for large targets while also supporting local localization of berry-level abnormal targets.

Second, the discriminative information of abnormal berries is local. According to the annotation rules in Section 2.1, the detection boxes for major and minor cover the visible region of a single grape berry, whereas local lesion-like or scar-like regions are not annotated separately. A detection box often contains a large amount of normal berry skin, waxy bloom, natural texture, or illumination reflection. If the network learns only the global appearance of the full bounding box, the local abnormal cues that are truly useful for category discrimination can easily be diluted by normal appearance responses.

To address these challenges, the proposed network progressively performs multi-scale feature fusion, local abnormal-cue enhancement, and semantic-guided recall compensation. EMBSFPN is adopted as the neck for multi-scale feature fusion, while IB-FLED and SGRCM are designed for local abnormal-cue enhancement and semantic-guided recall compensation, respectively.

### 2.3 Overall architecture of YEIS

YOLO11n was selected as the baseline because it is the smallest model in the YOLO11 family while retaining the three-scale P3–P5 detection hierarchy required for detecting both grape clusters and smaller abnormal berries. Nano-scale detectors are commonly used in agricultural vision to balance detection accuracy and computational cost (Li et al., 2025; Sun et al., 2025). Its publicly available configuration, pretrained weights, and unified training pipeline also facilitate reproducible and controlled ablation experiments (Jocher and Qiu, 2024; Ultralytics, 2026b). In YEIS, the detection head, loss function, and standard inference procedure were kept unchanged, and the modifications were restricted to feature fusion and refinement before the detection head.

YOLO-series detectors adopt multi-scale feature representations. In this study, the three detection scales output by the neck are denoted as P3, P4, and P5. P3 has relatively high spatial resolution and retains more small-target and local detail information. P4 provides a more balanced representation between spatial detail and semantic expression. P5 has a larger receptive field and provides high-level semantics and large-target context.

**Figure 2:**
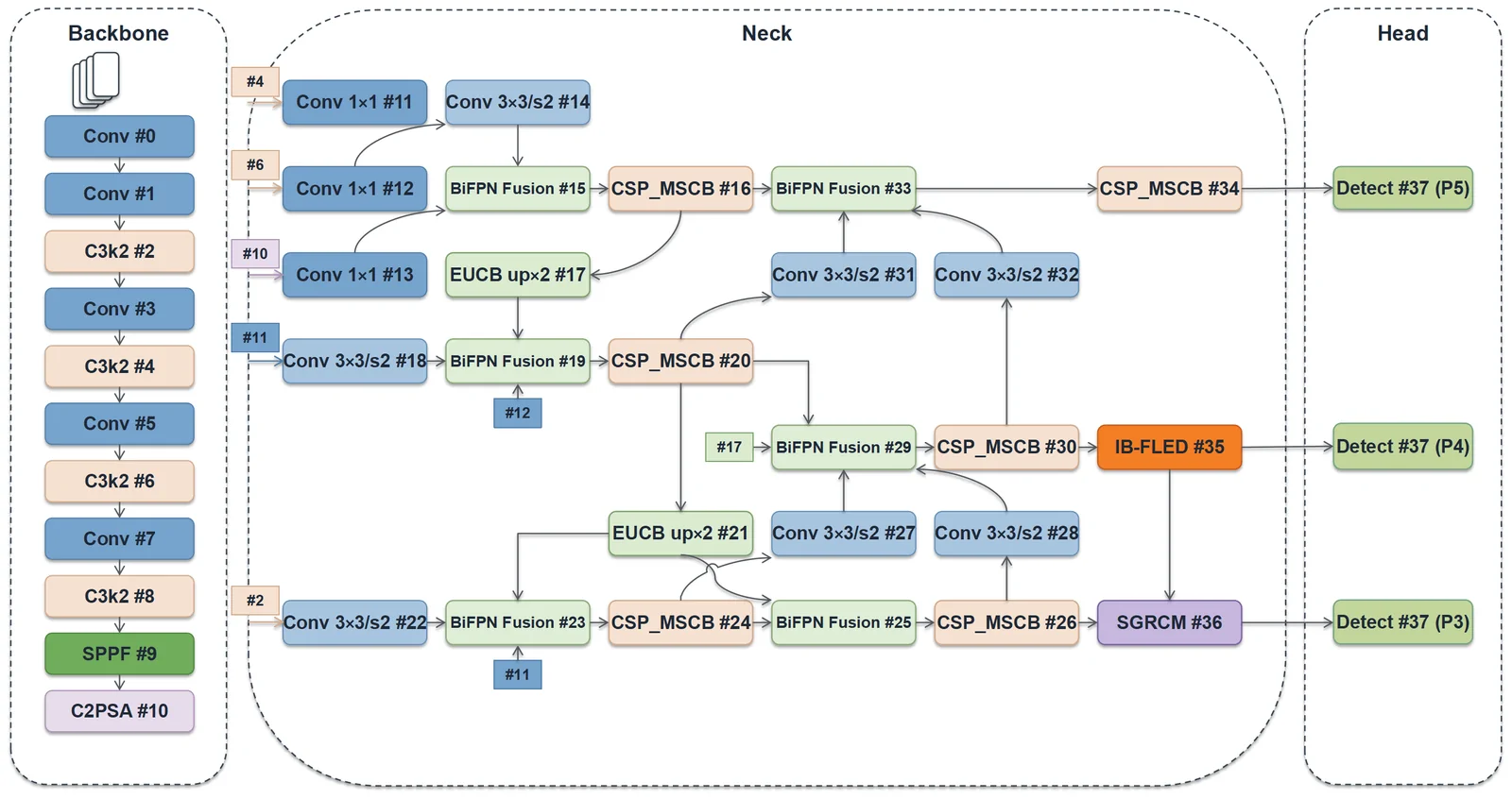
Overall architecture of YEIS.

As shown in **Figure 2**, EMBSFPN takes four backbone features, P2–P5, as inputs and constructs three candidate features at the P3, P4, and P5 scales, thereby alleviating the scale-representation discrepancy caused by the coexistence of cluster-level large targets and berry-level small- to medium-scale abnormal targets. The stride-4 P2 feature is downsampled to the P3 resolution by a 3 × 3 convolution with stride 2 and participates only in the initial P3 fusion node; it does not introduce a P2 detection output. IB-FLED is then inserted into the P4 branch after the EMBSFPN output to enhance local abnormal features within full abnormal-berry boxes. Finally, SGRCM uses the P4 semantic information refined by IB-FLED to compensate P3 detail candidates, guiding high-resolution details to focus more on abnormal berry-related regions.

Let the input image be *I*. The P2, P3, P4, and P5 features extracted from the backbone can be expressed as shown in Equation 1:

(1) $$\left\{F_{2},F_{3},F_{4},F_{5}\right\}=\text{Backbone}\left(I\right)$$

When the batch dimension and spatial dimensions are considered, the tensor form of the *k*-th level feature is given in Equation 2:

(2) $$F_{k}\in {\mathbb{R}}^{B\times C_{k}\times H_{k}\times W_{k}},\;k\in \left\{2,3,4,5\right\}$$

where *B* denotes the batch size; *F_k_* denotes the *P_k_*-level backbone feature; and *C_k_*, *H_k_*, and *W_k_* denote the number of channels, height, and width of the *k*-th scale feature map, respectively. EMBSFPN then fuses the four-level backbone features to produce three-scale candidate features as shown in Equation 3:

(3) $$\left\{F_{3}^{*},F_{4}^{*},F_{5}^{*}\right\}=\text{EMBSFPN}\left(F_{2},F_{3},F_{4},F_{5}\right)$$

The stride-4 feature *F*_2_ is downsampled and channel-aligned to the P3 resolution by a 3 × 3 convolution with stride 2, i.e., ${\hat{F}}_{2\to 3}=\phi_{2\to 3}\left(F_{2}\right)\in {\mathbb{R}}^{B\times C\times H_{3}\times W_{3}}$. It participates only in the initial P3 fusion node and does not produce a P2-scale detection output.

Based on the multi-scale candidate features, IB-FLED enhances local abnormal features on the P4 branch, as shown in Equation 4; SGRCM further updates the P3 detection input, as shown in Equation 5; and the final detection-head output is obtained as shown in Equation 6:

(4) $${\tilde{F}}_{4}=\text{IB‐FLED}\left(F_{4}^{*}\right)$$

(5) $${\tilde{F}}_{3}=\text{SGRCM}\left(F_{3}^{*},{\tilde{F}}_{4}\right)$$

(6) $$\hat{Y}=\text{Detect}\left({\tilde{F}}_{3},{\tilde{F}}_{4},F_{5}^{*}\right)$$

where $F_{3}^{*}$, $F_{4}^{*}$, and $F_{5}^{*}$ are the multi-scale features fused by EMBSFPN; ${\tilde{F}}_{4}$ is the P4 feature refined by IB-FLED; ${\tilde{F}}_{3}$ is the P3 feature compensated by SGRCM; and $\hat{Y}$ is the detection-head output. Equations 3-6 show that YEIS first establishes three-scale candidate features, then enhances intra-berry local abnormal features in the P4 branch, and finally uses refined P4 semantics to compensate P3 details. Detection prediction is ultimately completed based on ${\tilde{F}}_{3}$, ${\tilde{F}}_{4}$, and $F_{5}^{*}$. The specific module structures are described in Sections 2.3.1–2.3.3.

#### 2.3.1 EMBSFPN multi-scale feature fusion structure

**Table 2** shows a pronounced scale disparity among the detection classes. At an input size of 1280 × 1280, the median bounding-box dimensions is 191.2 × 297.5 pixels for GCLUSTER, compared with 52.0 × 61.1 and 70.2 × 75.7 pixels for major and minor, respectively; 88.72% of GCLUSTER instances are classified as large. Because abnormal berries occur within grape clusters, the neck must preserve fine spatial information while retaining cluster-level context. The standard YOLO11n neck mainly aggregates adjacent pyramid levels through concatenation, whereas EMBSFPN introduces aligned multi-level inputs and non-negative normalized branch weights at its fusion nodes (Gao et al., 2025). In our implementation, EMBSFPN takes four backbone features, P2–P5, as inputs and outputs three cross-scale candidate features at P3–P5 for the subsequent task-specific modules. The P3, P4, and P5 inputs are channel-aligned using 1 × 1 convolutions, whereas the stride-4 P2 input is downsampled and channel-aligned to the P3 resolution by a 3 × 3 convolution with stride 2 and participates only in the initial P3 fusion node. The aligned features are subsequently fused along bidirectional upsampling and downsampling paths. CSP_MSCB used scale-specific depthwise kernel sets of [1, 3, 5], [3, 5, 7], and [5, 7, 9] for P3, P4, and P5, respectively, while EUCB provided the upsampling operation. The suitability of this neck was evaluated against alternative fusion structures under the controlled protocol described in Section 3.2.1.

Let the input feature set of the *j*-th fusion node be defined as in Equation 7:

(7) $$X_{j}=\left\{X_{i}|i\in \Omega_{j}\right\}$$

where Ω*_j_* denotes the index set of input branches participating in the *j*-th fusion node, and *X_i_* denotes an input feature from a different scale or path. Because the spatial size and channel number of each input branch may differ, feature alignment is required before fusion through upsampling, downsampling, or convolutional mapping as shown in Equation 8:

(8) $${\hat{X}}_{i}=\phi_{i}\left(X_{i}\right),\;{\hat{X}}_{i}\in {\mathbb{R}}^{B\times C\times H_{j}\times W_{j}}$$

where $\phi_{i}\left(\cdot \right)$ denotes the scale-alignment and channel-mapping operation of the *i*-th input branch, ${\hat{X}}_{i}$ denotes the aligned input feature, and *C*, *H_j_*, and *W_j_* denote the channel number, height, and width of the *j*-th fusion node, respectively. The weights of the branches are normalized as defined in Equation 9:

(9) $$\alpha_{i}=\frac{\text{ReLU}\left(w_{i}\right)}{\sum_{m\in \Omega_{j}}\text{ReLU}\left(w_{m}\right)+\epsilon }$$

where *w_i_* is the learnable weight of the *i*-th input branch, *α_i_* is the normalized scale-contribution weight, and *ϵ* is a stability term. The output of the *j*-th fusion node can be expressed as shown in Equation 10:

(10) $$Y_{j}=N_{j}\left(\sum_{i\in \Omega_{j}}\alpha_{i}{\hat{X}}_{i}\right)$$

where $N_{j}\left(\cdot \right)$ denotes the feature integration unit after the fusion node, which corresponds to CSP_MSCB in this implementation, and *Y_j_* is the multi-scale fused feature output by the *j*-th fusion node. Finally, although EMBSFPN takes P2–P5 backbone features as inputs, it outputs candidate features only at the P3, P4, and P5 scales, which serve as the base features for the subsequent SGRCM, IB-FLED, and detection-head input pathways, respectively.

For the task considered in this study, EMBSFPN is not intended to directly discriminate abnormal berries. Instead, it first alleviates the scale-representation discrepancy caused by the coexistence of cluster-level large targets and berry-level small- to medium-scale abnormal targets. After this structure, the P3 branch retains high-resolution detail candidates, the P4 branch obtains a mid-level representation that combines spatial structure and semantic information, and the P5 branch provides high-level context with a larger receptive field.

#### 2.3.2 IB-FLED intra-berry local abnormal feature enhancement module

In this study, “intra-berry” refers to the visible portion of a single abnormal berry enclosed by a major or minor detection box. According to the annotation rules in Section 2.1, both major and minor use the visible abnormal berry region as the detection unit, and the local lesion-like or scar-like area inside the box is not annotated separately. This means that an abnormal berry box usually contains both normal appearance regions and local abnormal regions. If the network learns only the global appearance of the full box, local abnormal signals can be overwhelmed by normal appearance responses.

IB-FLED does not introduce additional ROI cropping or pixel-level supervision. Instead, it enhances feature responses related to local abnormalities in a residual manner on the P4 feature map. The module is connected to the P4 branch after the EMBSFPN output because P3 has high resolution but contains more shallow texture noise, whereas P5 has stronger semantics but lower spatial resolution. P4 provides a better balance between spatial structure and semantic stability and is therefore more suitable for intra-berry feature enhancement. The rationality of this insertion position is verified by ablation experiments in Section 3.2.4.

Let the input feature of IB-FLED be *X*. To reduce the computational cost of subsequent local modeling, a 1 × 1 convolution is first used for channel compression as shown in Equation 11:

(11) $$X_{r}=\phi_{r}\left(X\right),\;X_{r}\in {\mathbb{R}}^{B\times C_{r}\times H\times W}$$

where $\phi_{r}\left(\cdot \right)$ denotes the channel compression mapping, *X_r_* is the compressed input feature, and *C_r_* is the compressed channel number. A local average pooling operation is then used to estimate a low-frequency appearance reference as defined in Equation 12:

(12) $$X_{l}=A_{5\times 5}\left(X_{r}\right)$$

where $A_{5\times 5}\left(\cdot \right)$ denotes local average pooling with a 5 × 5 kernel, and *X_l_* is the low-frequency appearance estimate. The local residual is obtained from the difference between the compressed feature and the low-frequency appearance reference, as defined in Equation 13:

(13) $$R=X_{r}-X_{l}$$

where *R* is the local residual after removing the low-frequency appearance response. When a region is mainly covered by normal berry skin or smooth waxy bloom, the difference between *X_r_* and *X_l_* is relatively small. When lesion-like or scar-like local abnormalities are present, more pronounced difference responses appear at the corresponding positions in *R*.

Considering the morphological differences between the two types of abnormal appearances, IB-FLED constructs a line-shaped response branch, as defined in Equation 14, and a spot-like response branch, as defined in Equation 15, based on the local residual. The line-shaped response branch extracts directional structures through horizontal and vertical depthwise convolutions, whereas the spot-like response branch extracts local spot- or block-like responses through depthwise convolution and dilated depthwise convolution:

(14) $$S_{scar}=\phi_{s}\left(\left[D_{h}\left(R\right),D_{v}\left(R\right)\right]\right)$$

(15) $$S_{spot}=\phi_{p}\left(R\right)$$

where $D_{h}\left(\cdot \right)$ and $D_{v}\left(\cdot \right)$ denote horizontal and vertical depthwise convolutions, respectively; $\phi_{s}\left(\cdot \right)$ denotes the line-shaped response fusion convolution; $\phi_{p}\left(\cdot \right)$ denotes the spot-like response convolution; *S_scar_* and *S_spot_* denote the scar-like morphology response and lesion-like morphology response, respectively; and [·] denotes concatenation along the channel dimension.

To generate a local abnormal feature map at spatial positions, the two morphology responses and residual statistical responses are jointly combined into a feature vector as shown in Equation 16:

(16) $$Q=\left[S_{scar},S_{spot},{\text{Avg}}_{c}\left(R\right),{\text{Max}}_{c}\left(R\right),{\text{Avg}}_{c}\left(\left|R\right|\right)\right]$$

Based on *Q*, a spatial gating map is then generated, as defined in Equation 17:

(17) $$m=\sigma \left(\phi_{m}\left(Q\right)\right),\;m\in {\left[0,1\right]}^{B\times 1\times H\times W}$$

where Avg*_c_*(·) and Max*_c_*(·) denote average pooling and max pooling along the channel dimension, respectively; |·| denotes the absolute value operation; $\phi_{m}\left(\cdot \right)$ denotes the feature-map generation convolution; σ(·) denotes the Sigmoid function; and *m* is the local abnormal feature weight at each spatial position.

Based on *m*, the compressed feature is softly separated to obtain the abnormal feature-related response, as defined in Equation 18, and the normal appearance reference response, as defined in Equation 19:

(18) $$X_{e}=m⊙X_{r}$$

(19) $$X_{n}=\left(1-m\right)⊙X_{r}$$

The difference between them is given in Equation 20:

(20) $$\Delta X=X_{e}-X_{n}$$

where ⊙ denotes element-wise multiplication, *X_e_* is the abnormal feature-related response, *X_n_* is the normal appearance reference response, and Δ*X* denotes the difference between them. Finally, the abnormal feature response, difference feature, and two morphology responses are fused to obtain an enhanced residual, as defined in Equation 21, which is then injected into the original input feature in a residual manner:

(21) $$E=\phi_{o}\left(\phi_{f}\left(\left[X_{e},\Delta X,S_{scar},S_{spot}\right]\right)\right)$$

(22) $$Y=X+\gamma_{f}⊙E,\;\gamma_{f}\in {\mathbb{R}}^{1\times C\times 1\times 1}$$

where $\phi_{f}\left(\cdot \right)$ denotes the feature fusion convolution, $\phi_{o}\left(\cdot \right)$ denotes the channel restoration convolution, *E* is the enhanced residual, *γ_f_* is a learnable residual scaling coefficient, and *Y* is the output feature of IB-FLED. *γ_f_* participates in training with a small initial value, allowing the module to gradually inject local abnormal enhancement responses while preserving the stability of the original P4 semantic representation.

**Figure 3:**
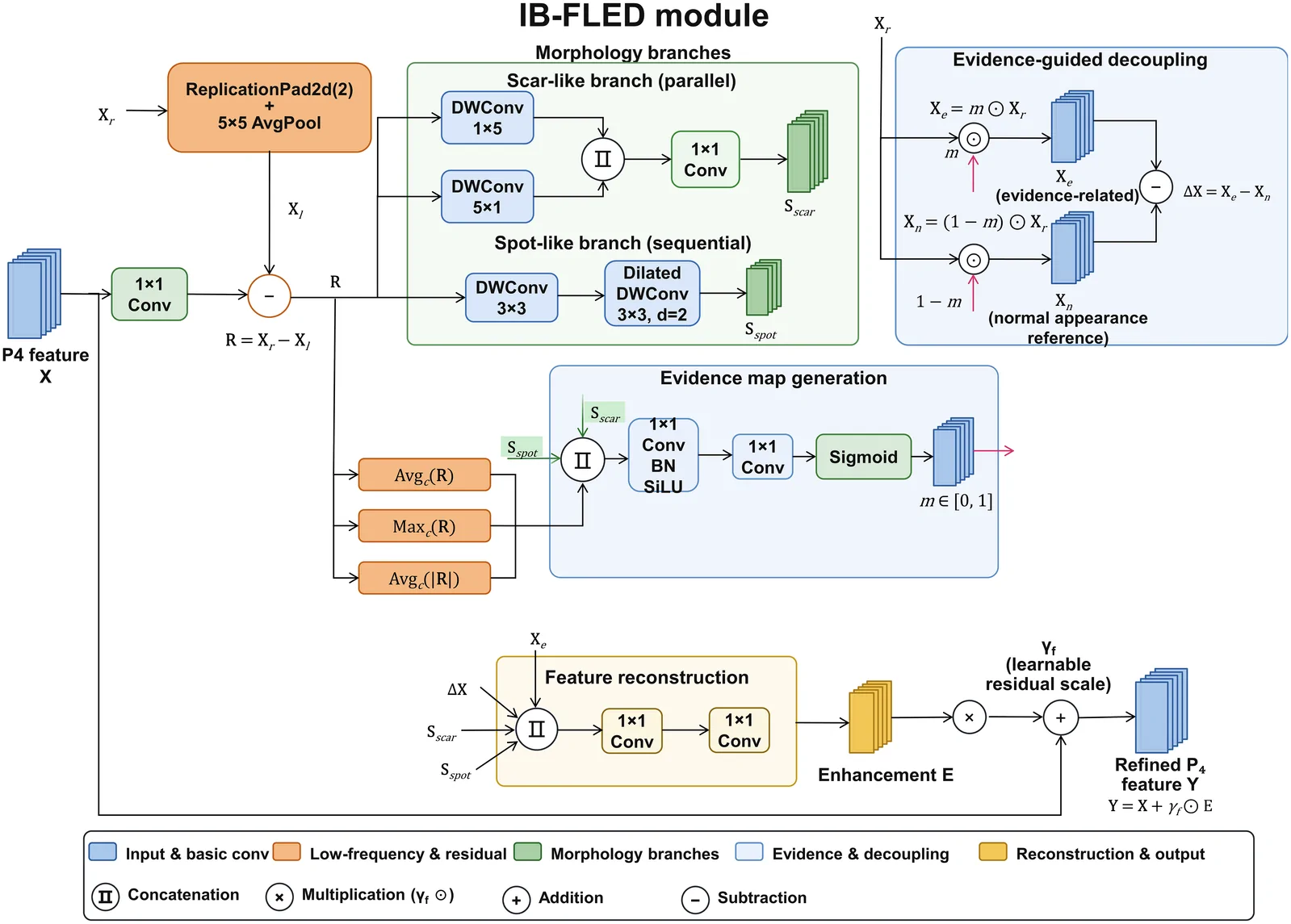
Structure of the IB-FLED module.

As indicated by (Equations 11-22) and **Figure 3**, without changing the spatial size or channel number of the P4 feature, IB-FLED enhances local feature information within a single abnormal-berry detection box through low-frequency appearance estimation, local residual modeling, morphology response extraction, and soft feature separation.

#### 2.3.3 SGRCM semantic-guided recall compensation module

The localization of berry-level abnormal targets depends on the high-resolution spatial details of the P3 layer. However, P3 is also more susceptible to shallow interference from waxy bloom, specular highlights, adjacent berry boundaries, and leaf textures. Directly enhancing P3 details may amplify real abnormal regions and irrelevant textures simultaneously. In contrast, the mid-level semantics of P4 are more stable, and after refinement by IB-FLED, local features associated with abnormal berries are further highlighted. Based on this observation, SGRCM is designed to use the refined P4 semantics to impose gated constraints on P3 detail candidates, making detail compensation more concentrated in abnormal berry-related regions.

Let the P3 feature output by EMBSFPN be $F_{3}^{*}$ and the P4 feature output by IB-FLED be ${\tilde{F}}_{4}$. SGRCM first performs channel mapping on the P3 feature, as shown in Equation 23, and then extracts detail candidate features, as defined in Equation 24:

(23) $$F_{3r}=\phi_{3r}\left(F_{3}^{*}\right)$$

(24) $$D_{3}=\phi_{d}\left(F_{3r}\right)$$

where $\phi_{3r}\left(\cdot \right)$ denotes the P3 channel mapping operation, $\phi_{d}\left(\cdot \right)$ denotes the detail candidate extraction operation, and *D*_3_ is the P3-layer detail candidate feature. Meanwhile, ${\tilde{F}}_{4}$ subjected to channel mapping and scale alignment so that its spatial size is consistent with the P3 feature, as shown in Equation 25:

(25) $$S_{4}=U\left(\phi_{4r}\left({\tilde{F}}_{4}\right)\right)$$

where $\phi_{4r}\left(\cdot \right)$ denotes the P4 channel mapping operation, *U*(·) denotes nearest-neighbor upsampling, and *S*_4_ is the scale-aligned P4 semantic feature.

To establish an interaction relationship between shallow details and mid-level semantics, SGRCM constructs a four-branch interaction tensor consisting of detail features, semantic features, consistency responses, and difference responses as shown in Equation 26:

(26) $$Z=\left[D_{3},S_{4},D_{3}⊙S_{4},\left|D_{3}-S_{4}\right|\right]$$

where $D_{3}⊙S_{4}$ denotes the consistency response between P3 details and P4 semantics, and $\left|D_{3}-S_{4}\right|$ denotes the difference response between them. A semantic-guided gating map is then generated from the interaction tensor, as defined in Equation 27:

(27) $$G=\sigma \left(\phi_{g}\left(Z\right)\right),\;G\in {\left[0,1\right]}^{B\times 1\times H_{3}\times W_{3}}$$

where $\phi_{g}\left(\cdot \right)$ denotes the gate-generation convolution, σ(·) is the Sigmoid function, and *G* is the semantic-guided gating weight at each spatial position. Based on *G*, selective compensation is applied to the P3 detail candidates, producing the final P3 feature input to the detection head as shown in Equation 28:

(28) $${\tilde{F}}_{3}=F_{3}^{*}+\gamma_{c}⊙\phi_{o}\left(G⊙D_{3}\right),\;\gamma_{c}\in {\mathbb{R}}^{1\times C_{3}\times 1\times 1}$$

where $\phi_{o}\left(\cdot \right)$ denotes the output mapping convolution, *γ*_c_ is a learnable residual scaling coefficient, and ${\tilde{F}}_{3}$ is the compensated P3 feature output by SGRCM. SGRCM updates only the P3 detection input and does not modify ${\tilde{F}}_{4}$, $F_{5}^{*}$, or the detection-head structure.

**Figure 4:**
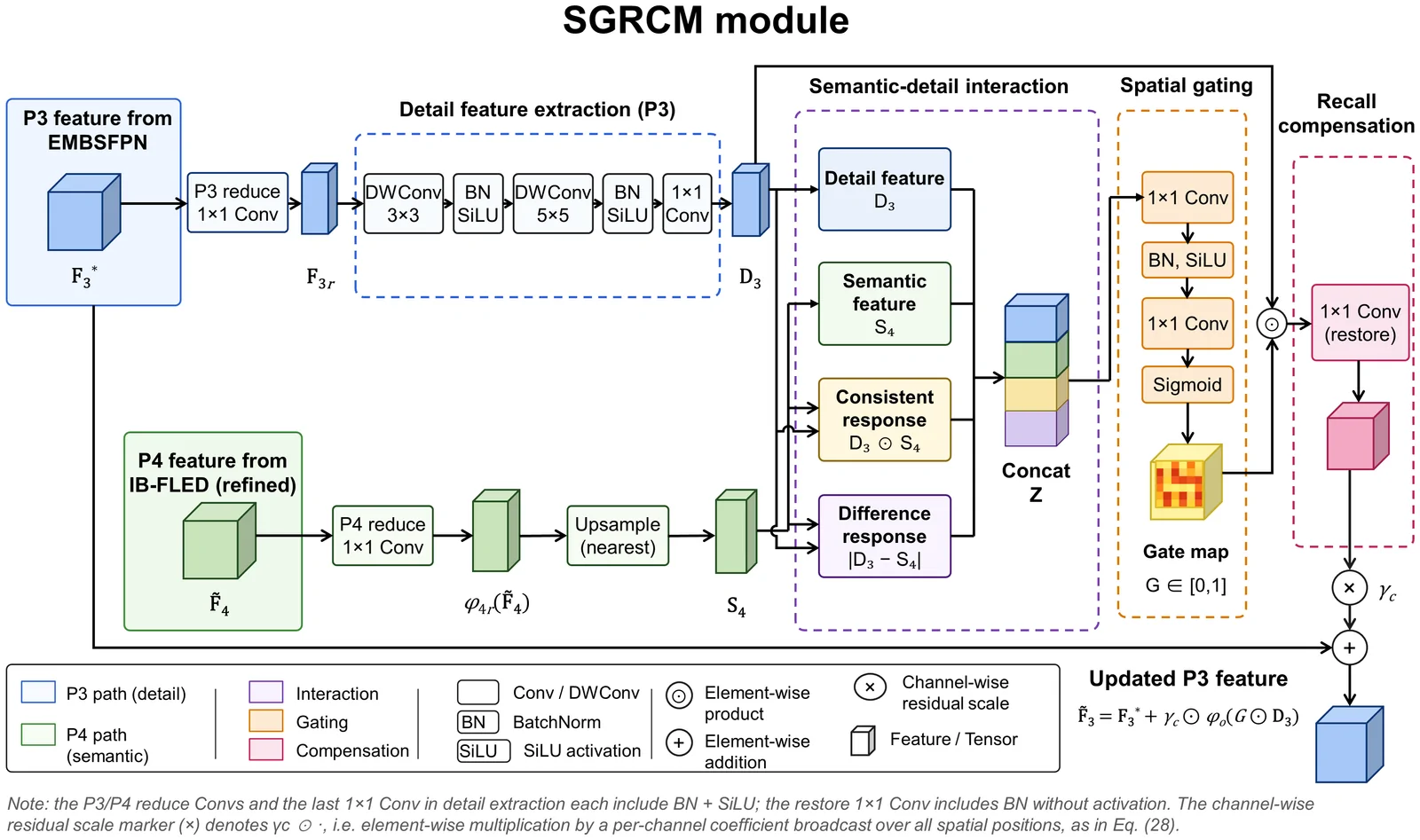
Structure of the SGRCM module.

As indicated by Equations 23-28 and **Figure 4**, SGRCM does not indiscriminately enhance shallow textures. Instead, it uses the P4 semantics refined by IB-FLED to spatially constrain P3 detail compensation, thereby reducing the risk of synchronously amplifying shallow interference. Its independent contribution and the role of P4 semantic gating are verified by ablation experiments in Section 3.2.5.

### 2.4 Training settings and evaluation metrics

All models were trained using the same dataset split and training protocol (**Table 3**). The input size was 1280 × 1280, the batch size was 16, and the maximum number of training epochs was 300, with an early-stopping patience of 100 epochs. SGD was used with an initial learning rate of 0.01, a final learning-rate factor of 0.01, momentum of 0.937, and weight decay of 0.0005. Training augmentation included HSV perturbation, translation, scaling, horizontal flipping, and Mosaic, with Mosaic disabled during the final 10 epochs. The complete training and augmentation settings are provided in **Table 3**. No data augmentation or test-time augmentation was applied to the validation or test sets.

**Table 3:** Experimental settings and training-parameter configuration.

Item	Setting
Baseline model	YOLO11n
Final model	YOLO11n + EMBSFPN + IB-FLED(P4) + SGRCM
Detection classes	GCLUSTER, major, minor
Training/validation/test images	1504/187/187
Hardware environment	32-core CPU; 120 GB memory; NVIDIA GeForce RTX 4090 GPU × 1
Software environment	Linux; Python 3.10.14; PyTorch 2.1.0; CUDA 12.1 (cuDNN 8.9.2); Ultralytics 8.3.9
Input size	1280 × 1280
Maximum epochs/early stopping	300/patience 100 epochs
Batch size/data-loading workers	16/8
Optimizer	SGD
Initial learning rate	0.01
Final learning-rate factor	0.01
Warm-up settings	3 epochs; momentum 0.8; bias learning rate 0.1
Momentum	0.937
Weight decay	0.0005
Loss gains (box/cls/dfl)	7.5/0.5/1.5
Random seeds	42, 123, 2024
Training controls	deterministic=True; AMP=False; cache=False; multi_scale=False
Confidence threshold for visualization/fixed-threshold analysis	0.25
NMS/inference IoU threshold	0.70 for class-aware conventional YOLO NMS; default inference for end-to-end models
Data augmentation	HSV gains 0.015/0.70/0.40; translation 0.10; scale 0.50; horizontal flip 0.50; Mosaic 1.0, disabled for final 10 epochs
Disabled augmentation	Rotation, shear, perspective, vertical flip, BGR channel reversal, MixUp, Copy-Paste, and test-time augmentation
Validation/test augmentation	None

To characterize training variability, the main experiments were independently repeated using random seeds 42, 123, and 2024, and the results are reported as the mean ± sample standard deviation (n = 3). A confidence threshold of 0.25 was used only for visualization and fixed-threshold error analysis. For detectors using conventional post-processing, including YOLOv5n, YOLOv8n, YOLOv9t, YOLO11n, YOLOv12n, and YEIS, class-aware non-maximum suppression (NMS) was applied at an IoU threshold of 0.70 (Neubeck and Van Gool, 2006; Ultralytics, 2026a). End-to-end detectors, including YOLOv10n, YOLO26n, and RT-DETR-R18, were evaluated using their default inference procedures without external NMS (Wang A. et al., 2024; Zhao et al., 2024; Jocher et al., 2026).

Training and evaluation were performed using a 32-core CPU, 120 GB of memory, and one NVIDIA GeForce RTX 4090 GPU. The software environment comprised Linux, Python 3.10.14, PyTorch 2.1.0, CUDA 12.1, cuDNN 8.9.2, and Ultralytics 8.3.9. Cross-model comparisons were conducted only under seed 42 and were therefore treated as descriptive comparisons without standard deviations or statistical inference. GFLOPs were calculated, and model-only inference FPS was measured, using an input size of 1280 × 1280.

The original YOLO11n detection loss was retained, as shown in Equation 29:

(29) $$ℒ=\lambda_{box}{ℒ}_{box}+\lambda_{cls}{ℒ}_{cls}+\lambda_{dfl}{ℒ}_{dfl}$$

Here, ${ℒ}_{\text{box}}$, ${ℒ}_{\text{cls}}$, and ${ℒ}_{\text{dfl}}$ denote the bounding-box regression loss, classification loss, and Distribution Focal Loss (Li et al., 2020), respectively. Their corresponding gains were set to 7.5, 0.5, and 1.5 (**Table 3**).

#### 2.4.1 Evaluation metrics and statistical analysis

Detection performance was evaluated using precision (P), recall (R), F1-score, average precision (AP), mAP_50_, mAP_75_, and mAP_50–95_ following the COCO-style evaluation protocol implemented in Ultralytics 8.3.9 (Lin et al., 2014). For P, R, and F1, predictions and ground-truth boxes of the same class were matched one-to-one at an IoU threshold of 0.50. Matched predictions were counted as true positives (TP), unmatched predictions as false positives (FP), and unmatched ground-truth boxes as false negatives (FN). This evaluation threshold was independent of the NMS IoU threshold of 0.70 used during inference.

Precision is calculated using Equation 30:

(30) $$P=\frac{\text{TP}}{\text{TP}+\text{FP}}$$

Recall is calculated using Equation 31:

(31) $$R=\frac{\text{TP}}{\text{TP}+\text{FN}}$$

The F1-score is calculated using Equation 32:

(32) $$F_{1}=\frac{2PR}{P+R}$$

For each run, the reported P, R, and F1 values were obtained at the confidence threshold that maximized the smoothed class-mean F1 curve and were then averaged over GCLUSTER, major, and minor.

For class c, AP was calculated as the area under the interpolated precision–recall curve, as shown in Equation 33:

(33) $${\text{AP}}_{c}\left(\tau \right)=\int_{0}^{1}P_{c}\left(R;\tau \right)\;\text{d}R$$

where τ denotes the evaluation IoU threshold. The mean AP over the three detection classes (C = 3) was calculated using Equation 34:

(34) $$\text{mAP}\left(\tau \right)=\frac{1}{C}\sum_{c=1}^{C}{\text{AP}}_{c}\left(\tau \right)$$

The mAP_50_ and mAP_75_ metrics correspond to IoU thresholds of 0.50 and 0.75, respectively. The mAP_50–95_ metric was obtained by averaging mAP over ten IoU thresholds from 0.50 to 0.95 at increments of 0.05.

Overall mAP_50–95_ was used as the principal comparison metric. Seed-matched differences between YOLO11n and YEIS were evaluated using a two-sided paired t-test (α = 0.05), and a 95% confidence interval was calculated for the mean paired difference using Student’s t distribution.

## 3 Results

### 3.1 Validation performance during training

**Figure 5:**
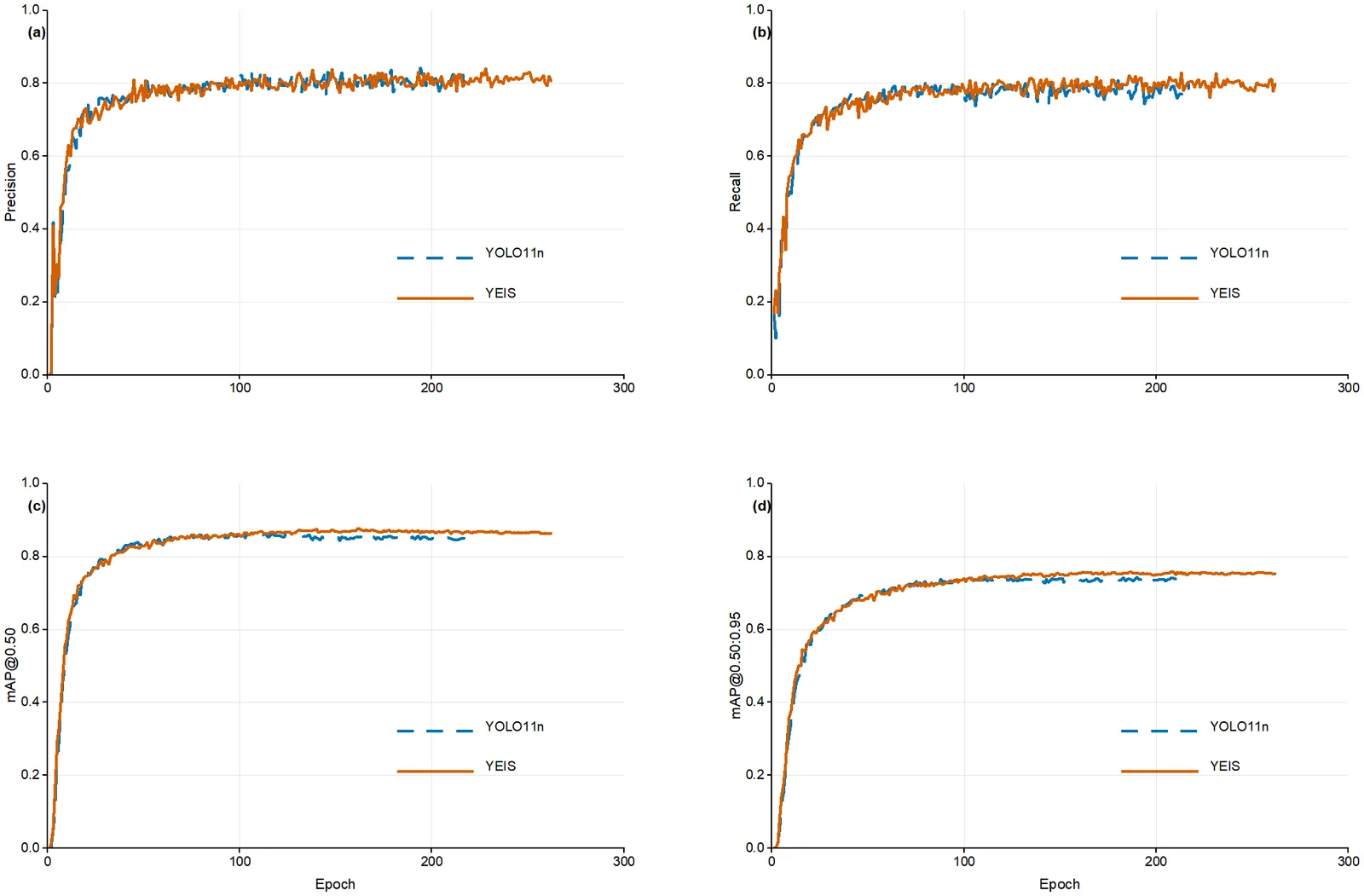
Validation performance of YOLO11n and YEIS during the seed-42 training runs: **(a)** precision; **(b)** recall; **(c)** mAP_50_; and **(d)** mAP_50–95_. The curves show unsmoothed epoch-wise values and terminate at the corresponding early-stopping epochs.

**Figure 5** presents the validation precision, recall, mAP_50_, and mAP_50–95_ for the representative seed-42 runs of YOLO11n and YEIS. The validation metrics increased rapidly during the early training stage and subsequently approached stable levels. In the later epochs, YEIS generally achieved higher recall and mAP values, while its precision remained comparable to that of YOLO11n. The curves terminate after 217 and 262 epochs for YOLO11n and YEIS, respectively, owing to early stopping.

### 3.2 Ablation experiments and controlled comparisons

#### 3.2.1 Controlled comparison of alternative neck architectures

To assess the suitability of different neck architectures for the present task, BiFPN (Tan et al., 2020), the RCNet neck (Zong et al., 2021), MAFPN (Yang et al., 2025), and EMBSFPN (Gao et al., 2025) were separately integrated into the YOLO11n baseline. The RCNet and MAFPN necks were adapted to match the feature dimensions and detection head of YOLO11n. For each configuration, only the neck architecture was changed, while all other training and evaluation settings were held constant. Each configuration was independently trained using random seeds 42, 123, and 2024, and the test-set results are reported as the mean ± sample standard deviation in **Table 4**.

**Table 4:** Controlled comparison of alternative neck architectures on the YOLO11n baseline.

Configuration	P (%)	R (%)	mAP_50_ (%)	mAP_75_ (%)	mAP_50–95_ (%)	Params (M)	GFLOPs (1280 × 1280)
YOLO11n	**82.44 ± 0.24**	79.11 ± 0.55	87.13 ± 0.41	82.10 ± 0.62	75.19 ± 0.04	2.583	25.3
YOLO11n + BiFPN	82.01 ± 1.25	78.85 ± 1.04	86.76 ± 0.56	81.45 ± 0.60	74.65 ± 0.16	**1.741**	**20.0**
YOLO11n + RCNet neck	82.22 ± 0.46	78.75 ± 2.51	87.14 ± 0.55	82.22 ± 0.87	75.63 ± 0.49	2.273	22.0
YOLO11n + MAFPN	81.55 ± 2.84	79.77 ± 1.42	87.26 ± 0.28	82.51 ± 0.11	76.01 ± 0.02	2.595	33.7
YOLO11n + EMBSFPN	80.90 ± 2.18	**80.98 ± 2.38**	**87.68 ± 0.49**	**82.70 ± 0.12**	**76.12 ± 0.27**	2.123	26.2

Bold values indicate the best performance for each evaluation metric.

EMBSFPN yielded the highest mean recall and all three mAP measures, although its mean precision was lower than that of YOLO11n (80.90% vs. 82.44%). Relative to the original YOLO11n neck, EMBSFPN increased mAP_50–95_ by 0.93 percentage points and reduced the parameter count from 2.583 M to 2.123 M. MAFPN achieved a comparable mAP_50–95_ of 76.01 ± 0.02%, but required 2.595 M parameters and 33.7 GFLOPs. Considering its accuracy–complexity balance and P3–P5 output structure, EMBSFPN was selected as the neck architecture for the subsequent experiments.

#### 3.2.2 Main-module ablation experiments

To evaluate the contribution of the proposed components, EMBSFPN and IB-FLED were first assessed individually on YOLO11n, followed by the EMBSFPN + IB-FLED, EMBSFPN + SGRCM, and complete YEIS combinations (**Table 5**). EMBSFPN constructs multi-scale candidate features, IB-FLED enhances local abnormal cues on the P4 branch, and SGRCM uses P4 semantic information to guide P3 detail compensation.

**Table 5:** Results of main-module ablation experiments.

Config.	EMB	IB	SG	Precision (%)	Recall (%)	mAP_50_ (%)	mAP_75_ (%)	mAP_50–95_ (%)	major mAP_50–95_ (%)	minor mAP_50–95_ (%)	Params (M)
YOLO11n	–	–	–	82.44 ± 0.24	79.11 ± 0.55	87.13 ± 0.41	82.10 ± 0.62	75.19 ± 0.04	77.38 ± 0.18	71.43 ± 0.57	2.583
+EMBSFPN	✓	–	–	80.90 ± 2.18	**80.98 ± 2.38**	87.68 ± 0.49	82.70 ± 0.12	76.12 ± 0.27	78.30 ± 0.48	73.00 ± 0.81	**2.123**
+IB-FLED	–	✓	–	81.69 ± 1.44	79.36 ± 0.76	87.20 ± 0.59	81.92 ± 0.28	75.26 ± 0.72	76.70 ± 0.87	72.25 ± 1.27	2.635
+EMBSFPN+IB-FLED	✓	✓	–	81.94 ± 0.77	80.76 ± 0.71	87.88 ± 0.15	83.15 ± 0.16	76.75 ± 0.38	78.53 ± 0.72	74.33 ± 0.39	2.137
+EMBSFPN+SGRCM	✓	–	✓	**83.46 ± 2.85**	78.48 ± 2.73	87.65 ± 0.26	82.97 ± 0.16	76.53 ± 0.21	78.67 ± 0.52	73.78 ± 0.48	2.136
**YEIS**	✓	✓	✓	83.21 ± 1.48	79.97 ± 1.66	**88.34 ± 0.70**	**83.27 ± 0.58**	**76.91 ± 0.60**	**78.98 ± 0.26**	**74.84 ± 1.05**	2.149

EMB, IB, and SG denote EMBSFPN, IB-FLED, and SGRCM, respectively; (✓) indicates that the corresponding module is used. YEIS refers to YOLO11n + EMBSFPN + IB-FLED(P4) + SGRCM. Bold values indicate the best performance for each evaluation metric.

**Table 5** presents the main-module ablation results. YOLO11n achieved mAP50, mAP75, and mAP50–95 values of 87.13%, 82.10%, and 75.19%, respectively. Replacing the original neck with EMBSFPN reduced the parameter count from 2.583 M to 2.123 M and increased mAP75 and mAP50–95 to 82.70% and 76.12%, respectively.

When IB-FLED was added alone, overall mAP_50–95_ changed from 75.19% to 75.26%. When IB-FLED was combined with EMBSFPN, overall and minor-class mAP_50–95_ reached 76.75% and 74.33%, respectively. Under the tested configurations, IB-FLED therefore provided a larger gain when used after EMBSFPN than when used alone.

**Figure 6:**
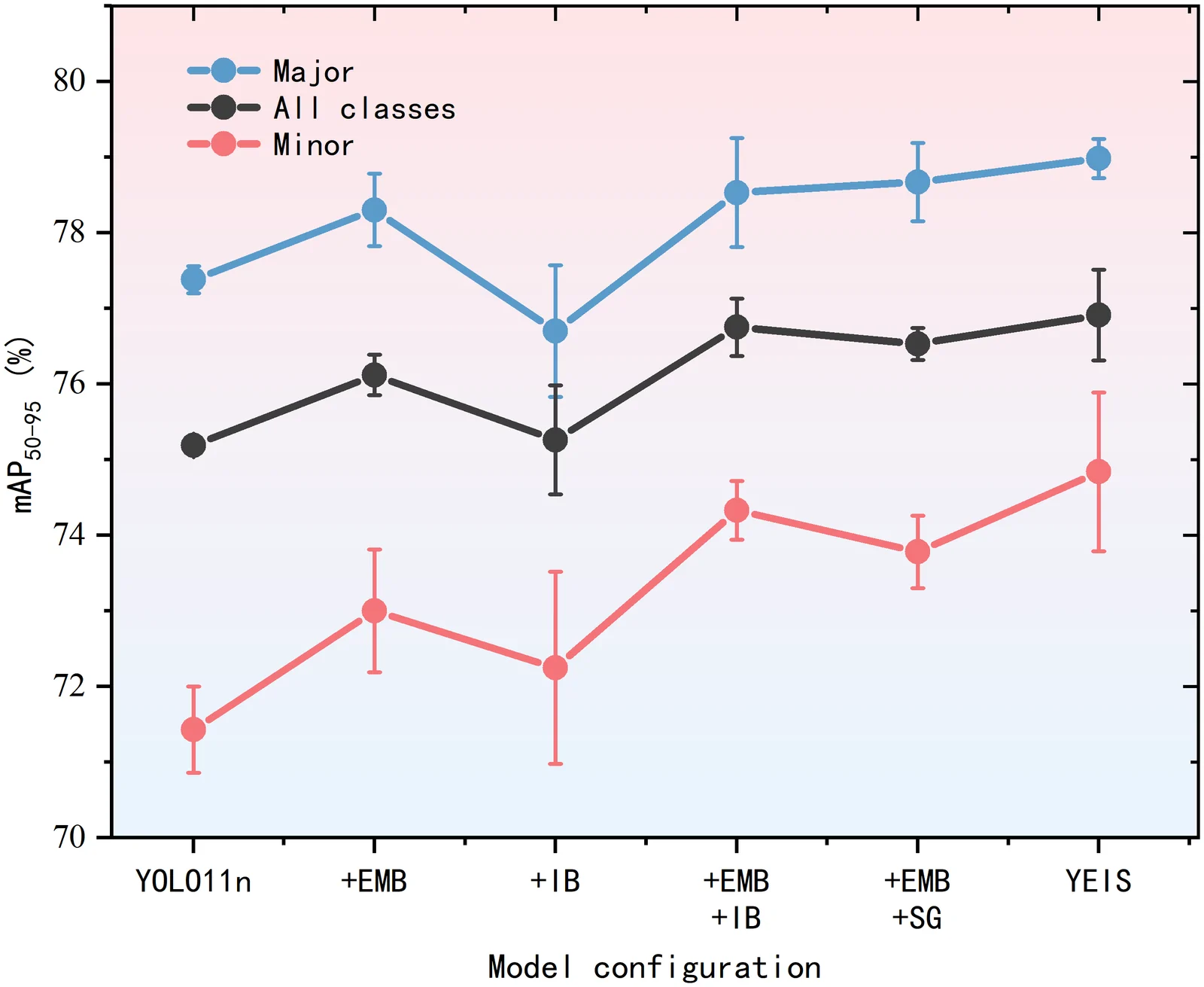
Changes in overall and abnormal-berry mAP_50–95_ in the main-module ablation experiments.

The complete YEIS model achieved the highest overall performance in **Table 5**, with mAP_50_, mAP_75_, and mAP_50–95_ values of 88.34%, 83.27%, and 76.91%, respectively. The major- and minor-class mAP_50–95_ values were 78.98% and 74.84%, respectively. **Figure 6** summarizes the changes in overall and abnormal-berry mAP_50–95_ across the module combinations.

#### 3.2.3 Class-wise gains of the baseline and complete model

The main-module ablation reflects the overall gains, but the benefits are not evenly distributed across target classes. To identify which target categories contribute most to the improvement of YEIS, the detection results of YOLO11n and the complete model on GCLUSTER, major, and minor were further compared (**Table 6**). The table reports all three classes under the same metric protocol to avoid selective reporting.

**Table 6:** Class-wise performance and seed-matched statistical comparison between YOLO11n and YEIS.

Class	Model	Precision (%)	Recall (%)	F1-score (%)	mAP_50_ (%)	mAP_75_ (%)	mAP_50–95_ (%)
**GCLUSTER**	YOLO11n	**88.27 ± 0.60**	**85.50 ± 1.00**	**86.86 ± 0.23**	**93.27 ± 0.09**	**84.19 ± 1.14**	76.76 ± 0.46
	YEIS	87.01 ± 1.48	85.05 ± 1.67	86.00 ± 0.21	92.96 ± 0.66	83.52 ± 0.65	**76.90 ± 0.79**
**major**	YOLO11n	**90.03 ± 1.79**	77.62 ± 1.80	83.34 ± 0.53	90.22 ± 0.72	85.50 ± 0.76	77.38 ± 0.18
	YEIS	89.15 ± 1.61	**79.25 ± 1.27**	**83.89 ± 0.28**	**91.13 ± 0.24**	**86.50 ± 0.28**	**78.98 ± 0.26**
**minor**	YOLO11n	69.02 ± 2.69	74.22 ± 2.68	71.46 ± 0.47	77.90 ± 0.80	76.62 ± 0.88	71.43 ± 0.57
	YEIS	**73.48 ± 2.16**	**75.62 ± 3.39**	**74.47 ± 0.53**	**80.94 ± 1.30**	**79.77 ± 1.10**	**74.84 ± 1.05**

Seed-matched comparison of overall mAP_50–95_						
Metric	YOLO11n (%)	YEIS (%)	Mean difference (95% CI), pp			Paired t-test p
Overall mAP_50–95_	75.19 ± 0.04	76.91 ± 0.60	+1.71 (0.20–3.23)			0.040

Values are mean ± SD over three matched random seeds. The mean difference and 95% CI are expressed in percentage points. The p-value is from a two-sided paired t-test. Bold values indicate the best performance for each evaluation metric.

Across the three matched runs, YEIS increased overall mAP_50–95_ from 75.19 ± 0.04% to 76.91 ± 0.60%, corresponding to a mean gain of 1.71 percentage points (95% CI: 0.20–3.23; two-sided paired t-test, p = 0.040). All three paired differences were positive.

The class-wise results were treated descriptively. YEIS increased mAP_50–95_ by 1.61 percentage points for major and 3.41 percentage points for minor, whereas GCLUSTER changed by 0.13 percentage points. The overall gain was therefore concentrated in the two abnormal-berry classes, while GCLUSTER performance remained similar.

#### 3.2.4 IB-FLED insertion position and internal mechanism analysis

IB-FLED was evaluated at different insertion positions on the EMBSFPN configuration, followed by component ablations of its P4 implementation. **Table 7** reports both analyses under the same metric protocol.

**Table 7:** Ablation results for the insertion position and components of IB-FLED.

Ablation type	Configuration	mAP_50_ (%)	mAP_75_ (%)	mAP_50–95_ (%)	major mAP_50–95_ (%)	minor mAP_50–95_ (%)
Insertion position	EMBSFPN	87.68 ± 0.49	82.70 ± 0.12	76.12 ± 0.27	78.30 ± 0.48	73.00 ± 0.81
	IB-FLED at P3	**88.01 ± 0.05**	82.63 ± 0.30	76.24 ± 0.10	**78.55 ± 0.34**	73.98 ± 0.37
	IB-FLED at P4	87.88 ± 0.15	**83.15 ± 0.16**	**76.75 ± 0.38**	78.53 ± 0.72	**74.33 ± 0.39**
	IB-FLED at P3+P4	87.35 ± 0.51	82.13 ± 0.71	75.89 ± 0.59	78.12 ± 0.10	72.86 ± 1.04
Internal mechanism	Complete IB-FLED(P4)	87.88 ± 0.15	**83.15 ± 0.16**	**76.75 ± 0.38**	78.53 ± 0.72	74.33 ± 0.39
	Without low-frequency appearance estimation and residual modeling	**88.01 ± 0.33**	82.91 ± 0.38	76.54 ± 0.50	78.37 ± 1.00	73.89 ± 0.49
	Without morphology-aware response branches	87.77 ± 0.10	83.04 ± 0.24	76.41 ± 0.35	**78.59 ± 0.23**	73.75 ± 0.75
	Without local abnormal-response difference modeling	87.64 ± 0.47	82.71 ± 0.85	76.25 ± 0.51	78.58 ± 0.50	73.68 ± 1.13
	Without local abnormal-response map	87.96 ± 0.47	82.83 ± 0.43	76.52 ± 0.33	78.39 ± 0.52	**74.35 ± 0.17**

All component ablations use EMBSFPN + IB-FLED(P4) as the complete configuration. Bold values indicate the best performance for each evaluation metric.

IB-FLED at P3 yielded a slightly higher mAP_50_ than at P4 (88.01% vs. 87.88%), whereas P4 produced higher mAP_75_, overall mAP_50–95_, and minor-class mAP_50–95_. Using both P3 and P4 did not provide an additional benefit: overall mAP_50–95_ decreased to 75.89%, below both P4-only insertion (76.75%) and the EMBSFPN baseline (76.12%). Because feature redundancy was not directly measured, no specific mechanism is assigned to this decrease. Overall, P4 provided the strongest results for the metrics emphasized in this task.

For the P4 component ablations, complete IB-FLED achieved overall and minor-class mAP_50–95_ values of 76.75% and 74.33%, respectively. Removing low-frequency appearance estimation and residual modeling reduced these values to 76.54% and 73.89%; removing the morphology-aware branches reduced them to 76.41% and 73.75%; and removing local abnormal-response difference modeling reduced them to 76.25% and 73.68%. Major-class mAP_50–95_ varied only slightly across these settings.

#### 3.2.5 SGRCM semantic-gating and module-combination analysis

To evaluate SGRCM and P4 semantic gating, four configurations were compared: EMBSFPN, EMBSFPN + SGRCM, complete YEIS, and YEIS without P4 semantic gating (**Table 8**).

**Table 8:** Ablation results for SGRCM semantic gating and module combinations.

Configuration	mAP_50_ (%)	mAP_75_ (%)	mAP_50–95_ (%)	major mAP_50–95_ (%)	minor mAP_50–95_ (%)
EMBSFPN	87.68 ± 0.49	82.70 ± 0.12	76.12 ± 0.27	78.30 ± 0.48	73.00 ± 0.81
EMBSFPN+SGRCM	87.65 ± 0.26	82.97 ± 0.16	76.53 ± 0.21	78.67 ± 0.52	73.78 ± 0.48
YEIS without P4 semantic gating	87.66 ± 0.36	82.56 ± 0.56	76.32 ± 0.53	78.34 ± 1.05	73.32 ± 0.48
**YEIS**	**88.34 ± 0.70**	**83.27 ± 0.58**	**76.91 ± 0.60**	**78.98 ± 0.26**	**74.84 ± 1.05**

Bold values indicate the best performance for each evaluation metric.

Adding SGRCM to EMBSFPN changed mAP_50_ from 87.68% to 87.65%, while mAP_75_ and mAP_50–95_ increased from 82.70% and 76.12% to 82.97% and 76.53%, respectively. The larger gains at stricter evaluation IoU thresholds are consistent with improved localization quality.

Complete YEIS achieved overall, major-class, and minor-class mAP_50–95_ values of 76.91%, 78.98%, and 74.84%, respectively. Relative to YEIS without P4 semantic gating, overall and minor-class mAP_50–95_ were higher by 0.59 and 1.52 percentage points, respectively. These differences are consistent with a beneficial contribution of P4 semantic gating in the complete configuration.

### 3.3 Detection results and error analysis on complex field samples

#### 3.3.1 Class-wise missed-detection statistics on the full test set

Field images of Munage grapes usually contain multiple interfering factors simultaneously, including color-similar backgrounds, dense berries, branch and leaf occlusion, overlapping clusters, specular highlights, and locally weak boundaries. To analyze model performance on complex samples from the perspective of error types, TP, FN, FP, and class-wise missed detections were counted based on the prediction results for the full test set, and typical complex-sample visualizations were further used for explanation. The results are shown in **Table 9**.

**Table 9:** Class-wise missed detections and abnormal-berry missed detections.

Model	TP	FN	FP	GCLUSTER FN	major FN	minor FN	Abnormal berry FN
YOLO11n	1673	267	646	55	125	87	212
EMBSFPN	1701	239	702	**43**	112	84	196
EMBSFPN+IB-FLED	1665	275	633	63	128	84	212
EMBSFPN+SGRCM	1680	260	**626**	68	118	74	192
**YEIS**	**1732**	**208**	768	54	**100**	**54**	**154**

Counts were obtained from the seed-42 best checkpoints on the full 187-image test set at a confidence threshold of 0.25, using class-aware NMS at an IoU threshold of 0.70 followed by same-class one-to-one matching at an evaluation IoU threshold of 0.50. Abnormal berry FN is the sum of major FN and minor FN. Bold values indicate the best performance for each evaluation metric.

In **Table 9**, the overall FN of YOLO11n is 267, whereas that of YEIS decreases to 208, corresponding to 59 fewer missed targets. However, the class-wise reduction is uneven: GCLUSTER decreases only from 55 to 54, showing almost no change, while major decreases from 125 to 100 and minor decreases from 87 to 54. The combined FN of abnormal berries decreases from 212 to 154, accounting for nearly all of the reduction. This indicates that YEIS brings little improvement in suppressing missed detections for large grape-cluster targets, and the improvement is mainly concentrated on scar-like and lesion-like abnormal berries. Meanwhile, the FP of YEIS increases from 646 to 768, approximately 122 more false positives, indicating that the model produces more false detections while improving abnormal-berry recall. Considering the field interference factors described in Section 2.2, normal berry-skin texture, waxy bloom coverage, specular highlights, and adjacent berry boundaries may still be misclassified as abnormal candidate regions. Overall, the main improvement of the proposed method lies in reducing missed detections and improving localization accuracy for abnormal berries, while false-positive suppression remains a direction worthy of further optimization.

#### 3.3.2 Visualization of detection results on typical complex samples

**Figure 7:**
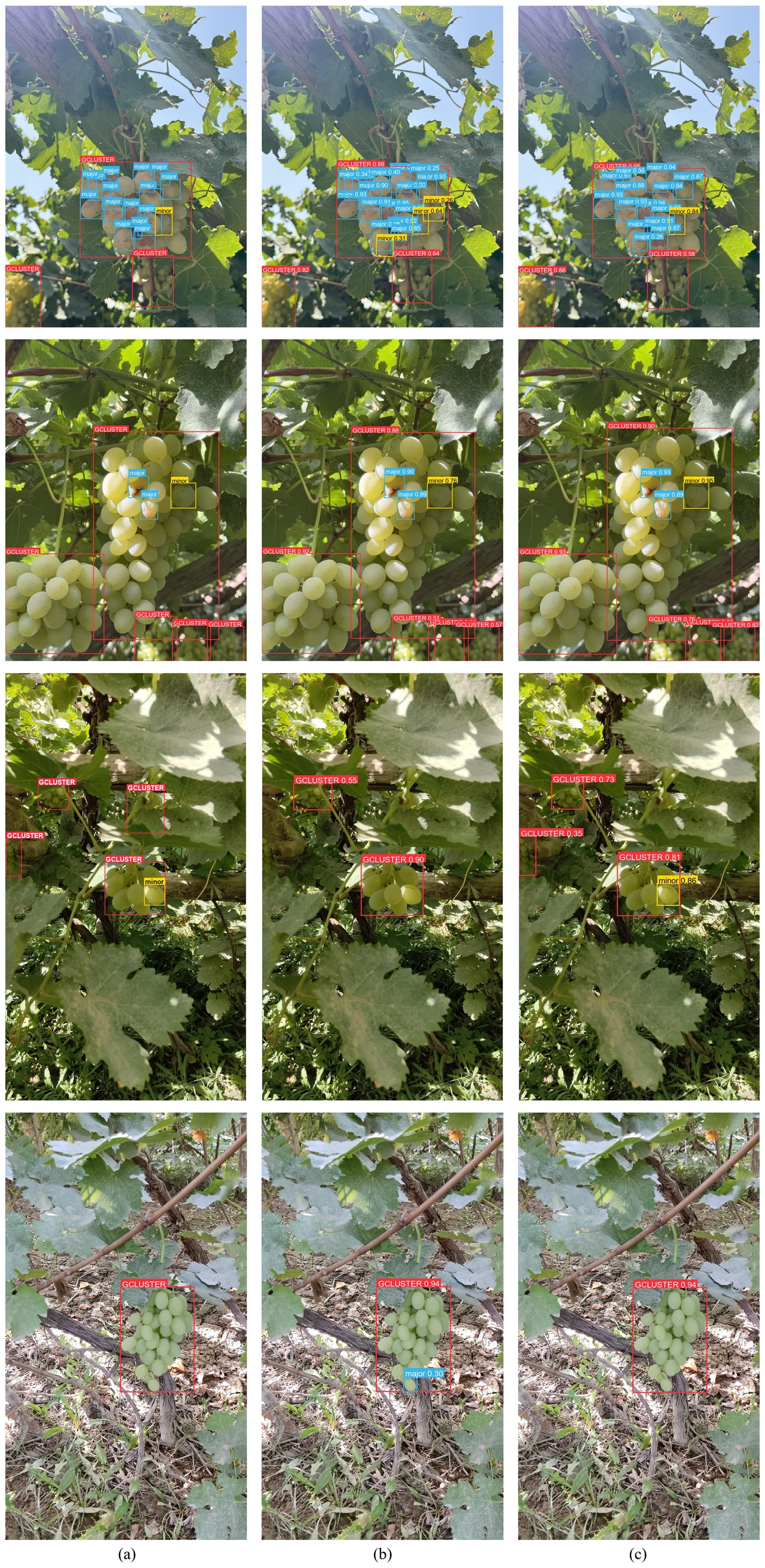
Comparison of annotations, YOLO11n detections, and YEIS detections in typical complex field samples: **(a)** annotations; **(b)** YOLO11n; **(c)** YEIS.

To visualize the reduction in missed detections and the changes in false detections reported in **Table 9**, representative test samples with color-similar backgrounds, dense berries, specular highlights, overlapping clusters, and branch and leaf occlusion were selected. The annotations, YOLO11n detections, and YEIS detections were compared visually, as shown in **Figure 7**.

As shown in **Figure 7**, YOLO11n tends to miss abnormal berries in dense or heavily occluded samples, especially minor targets with weak scars and colors similar to normal berry skin. YEIS can additionally detect some abnormal berries missed by the baseline and can maintain simultaneous detection of grape clusters and abnormal berries in dense multi-target regions. This is consistent with the simultaneous reductions in major FN, minor FN, and abnormal-berry FN shown in **Table 9**. In addition, some normal berry-skin textures or strong reflective regions are also misclassified as abnormal berries, corresponding to the increase in FP shown in **Table 9**.

#### 3.3.3 Heatmap response analysis

**Figure 8:**
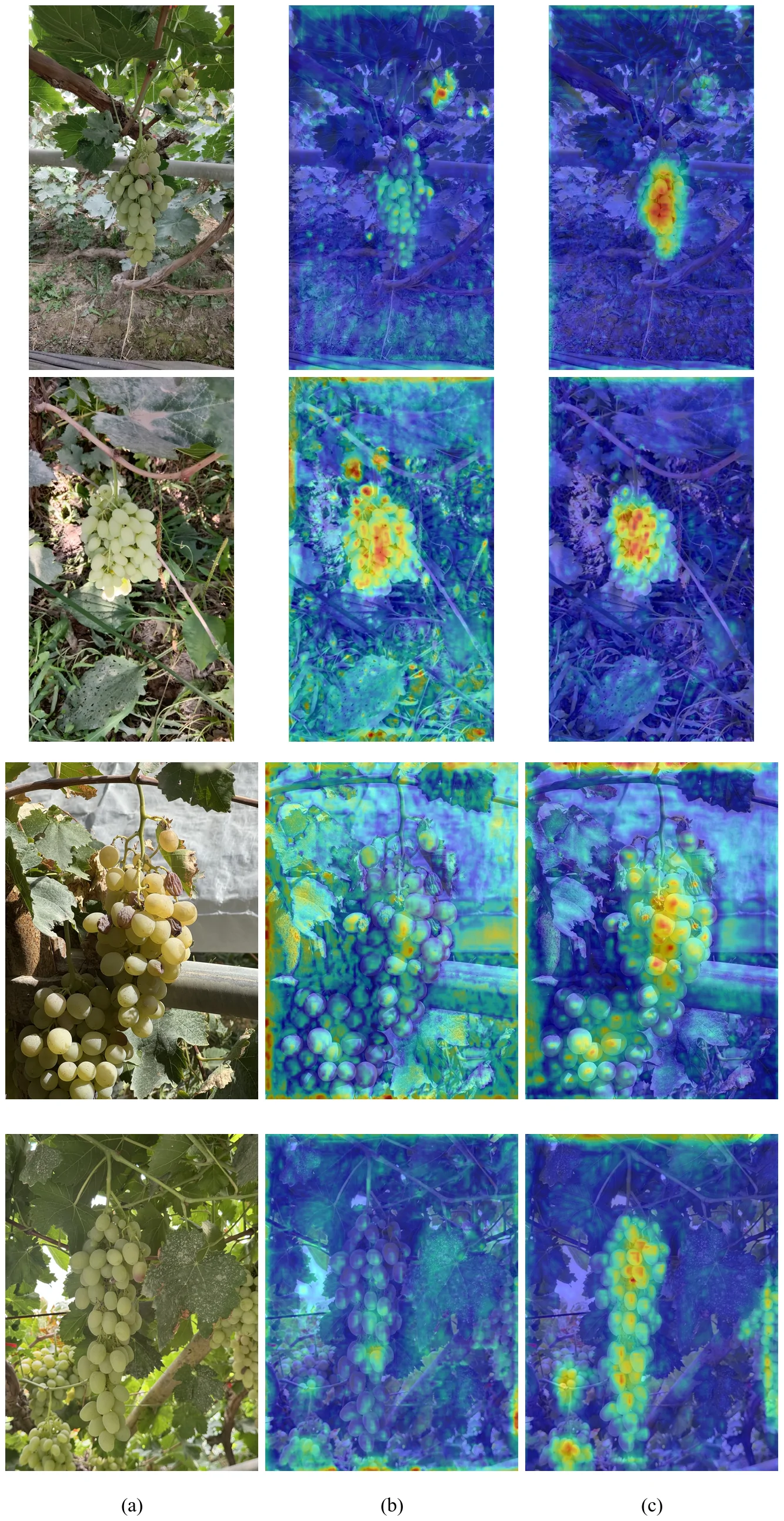
Grad-CAM++-based heatmap response comparison between YOLO11n and YEIS on typical samples: **(a)** original image; **(b)** YOLO11n; **(c)** YEIS.

**Figure 8** uses Grad-CAM++ (Chattopadhay et al., 2018) to visualize detection-related feature responses of YOLO11n and YEIS, allowing comparison of their spatial response differences on the same inputs. Warmer colors indicate stronger responses.

In **Figure 8**, YOLO11n responses extended to leaves, branches, cluster contours, and background highlights in several samples, whereas YEIS responses were more concentrated around abnormal berries and their neighboring regions. This qualitative pattern is consistent with more localized response maps in YEIS; however, Grad-CAM++ does not establish the causal contribution of individual modules and is not a substitute for quantitative evaluation.

### 3.4 Comparison with representative object detection models

#### 3.4.1 Comparison of accuracy, speed, and parameter size

To evaluate the performance differences between YEIS and representative detectors, YOLOv5n, YOLOv8n, YOLOv9t, YOLOv10n, YOLO11n, YOLOv12n, YOLO26n, and RT-DETR-R18 were selected for cross-model comparison under the same dataset split (Jocher, 2020; Jocher et al., 2023; Wang C.-Y. et al., 2024, Wang A. et al., 2024; Jocher and Qiu, 2024; Zhao et al., 2024; Tian Y. et al., 2025; Jocher et al., 2026; Ultralytics, 2026b). Unlike the preceding ablation experiments, this section uses only seed = 42, and standard deviations are not reported in **Table 10**. The experiment is intended only as a representative comparison of different detectors under the same protocol and is not used for statistical inference.

**Table 10:** Cross-model comparison of different object detection models on the test set.

Model	Params (M)	GFLOPs (1280 × 1280)	FPS	P (%)	R (%)	F1 (%)	mAP_50_ (%)	mAP_75_ (%)	mAP_50–95_ (%)
YOLOv5n	2.504	28.3	**378.53**	83.40	77.82	80.38	86.66	80.20	73.38
YOLOv8n	3.006	32.3	378.41	**84.34**	77.09	80.47	86.48	81.30	74.15
YOLOv9t	**1.971**	30.4	285.34	83.45	78.00	80.57	87.04	82.09	75.39
YOLOv10n	2.266	26.1	366.71	80.97	78.05	79.44	84.64	79.15	72.86
YOLO11n	2.583	25.3	252.37	82.72	78.66	80.60	87.11	81.63	75.15
YOLOv12n	2.557	25.3	222.73	81.90	79.33	80.52	87.51	82.18	75.07
YOLO26n	2.375	**20.8**	324.21	81.60	75.39	78.33	84.15	77.60	71.12
RT-DETR-R18	19.876	222.5	82.67	84.31	78.79	81.42	86.23	80.73	74.71
**YEIS**	2.149	26.9	206.44	82.51	**81.13**	**81.66**	**88.77**	**83.62**	**77.06**

All models use the same training/validation/test split, 1280 × 1280 input size, and unified test-set evaluation script for accuracy evaluation. GFLOPs are calculated at a 1280 × 1280 reference input and are used only for relative computational-complexity comparison. Post-processing follows the default inference pipeline of each model. FPS is measured during model inference at the 1280 × 1280 input size. Bold values indicate the best performance for each evaluation metric.

Under seed 42, YEIS achieved the highest Recall, mAP_50_, mAP_75_, and mAP_50–95_ among the compared models, reaching 81.13%, 88.77%, 83.62%, and 77.06%, respectively. Relative to YOLO11n, YEIS increased mAP_50–95_ by 1.91 percentage points while reducing the parameter count from 2.583 M to 2.149 M. Its mAP_50–95_ was also 1.67 percentage points higher than that of YOLOv9t, the strongest alternative model for this metric.

#### 3.4.2 Accuracy-speed-parameter-size trade-off analysis

**Figure 9:**
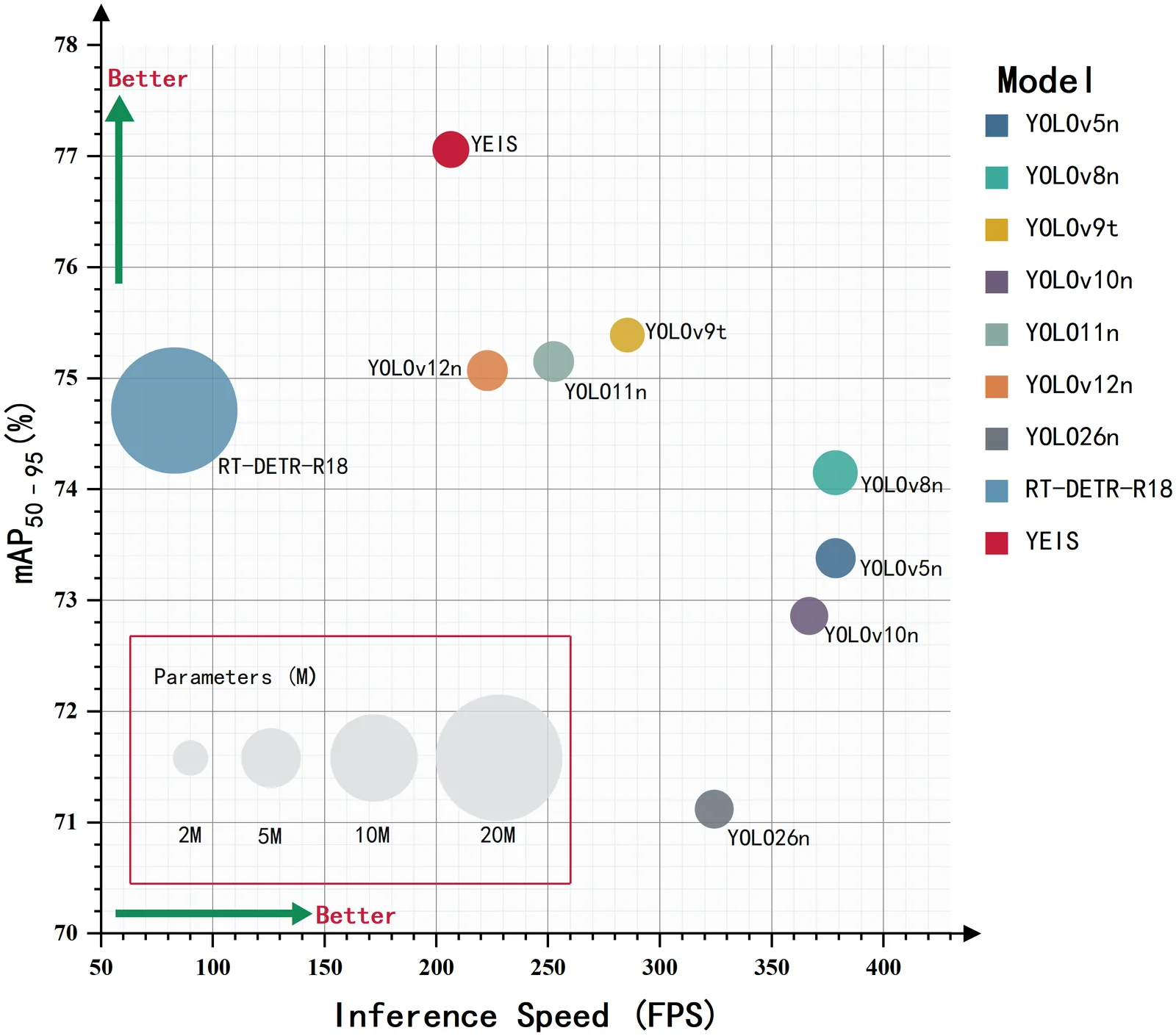
Relationship among accuracy, speed, and parameter size of the comparative models under seed 42.

To more intuitively present the relationships among accuracy, speed, and parameter size for different detectors, an mAP_50–95_ versus FPS bubble plot was drawn, where the horizontal axis represents inference speed, the vertical axis represents mAP_50–95_, and bubble size indicates the relative parameter count, as shown in **Figure 9**.

**Figure 9** shows that YEIS is located in a relatively high mAP_50–95_ region, although its FPS is lower than those of higher-throughput models such as YOLOv5n, YOLOv8n, YOLOv10n, and YOLO26n. Its Precision is also lower than those of YOLOv8n and RT-DETR-R18. Therefore, the advantage of YEIS lies primarily in Recall, mAP_75_, and mAP_50–95_ rather than inference speed or Precision.

### 3.5 Comparison of feature-fusion and resampling methods

FreqFusion (Chen L. et al., 2024), HS-FPN (Shi et al., 2025), and DySample+ (Liu W. et al., 2023) were implemented within the YOLO11n framework and evaluated together with YOLO11n, YOLO11n + EMBSFPN, and YEIS. All configurations used the same dataset split, training protocol, and test settings. This experiment was conducted under seed 42; therefore, the results are presented as descriptive comparisons without statistical inference.

YEIS achieved the highest mAP_50_, mAP_75_, and mAP_50–95_ among the evaluated configurations, reaching 88.77%, 83.62%, and 77.06%, respectively. FreqFusion achieved the highest Precision (85.35%), EMBSFPN achieved the highest Recall (81.72%), and DySample+ achieved the highest inference speed (274.57 FPS).

Compared with FreqFusion, which achieved the highest mAP_50–95_ among the alternative configurations, YEIS improved mAP_50–95_ by 0.48 percentage points, used 0.355 M fewer parameters, and achieved a higher inference speed (206.44 vs. 191.52 FPS). YEIS also exceeded YOLO11n + EMBSFPN by 0.80 percentage points in mAP_50–95_.

Relative to YOLO11n, YEIS improved mAP_50–95_ by 1.91 percentage points and reduced the parameter count by 16.8%, from 2.583 M to 2.149 M. However, its inference speed decreased by 18.2%, from 252.37 to 206.44 FPS. Overall, YEIS achieved the highest mAP-based detection accuracy in this comparison while retaining a lower parameter count than YOLO11n, although this improvement was accompanied by reduced inference speed.

## 4 Discussion

### 4.1 Model advantages and underlying mechanisms

The task addressed in this study differs from simple grape-cluster detection, pathological cause diagnosis, or pixel-level lesion segmentation. Instead, it aims to simultaneously detect cluster-level GCLUSTER targets and berry-level abnormal targets, namely major and minor, in field images with color-similar backgrounds. The experimental results indicate that the main performance gains of YEIS do not arise from the detection of large grape-cluster targets, but are concentrated on berry-level abnormal targets, especially minor-class abnormal berries with weak boundaries and low contrast.

Although the absolute improvement in overall mAP_50–95_ was moderate, the class-wise results show that this average was moderated by the nearly unchanged GCLUSTER category. The larger improvements for the major and minor berry-level categories are consistent with the intended function of YEIS, which focuses on enhancing localized abnormal evidence within berries while maintaining cluster-level detection performance. Correspondingly, the reduction in missed detections was contributed almost entirely by abnormal berry targets rather than by large grape-cluster targets.

Recent agricultural detectors such as BHC-YOLOv8 and GCP-YOLO have demonstrated the value of strengthened pyramid fusion for field targets (Zhan et al., 2024; Kuang et al., 2026). In our controlled comparison (**Table 4**), EMBSFPN obtained the highest three-seed mean mAP_50–95_ but exceeded MAFPN by only 0.11 percentage points, while FreqFusion produced a higher point estimate than EMBSFPN under seed 42. EMBSFPN is therefore regarded as a competitive feature-fusion structure for this task rather than a universally superior neck. RCNet and MHAF-YOLO emphasize cross-scale information transfer (Zong et al., 2021; Yang et al., 2025), whereas FreqFusion and BPIM focus on frequency-aware alignment and boundary/position cues, respectively (Chen L. et al., 2024; Huang et al., 2026). YEIS differs in targeting localized abnormal evidence that occupies only part of a full-berry bounding box nested within a grape cluster: IB-FLED enhances this evidence on the P4 branch, whereas SGRCM constrains P3 detail compensation using the refined P4 semantics. The complete YEIS model achieved the highest mAP_50–95_ in the seed-42 comparison (**Table 11**), suggesting that these modules provide complementary benefits for the present task.

**Table 11:** Comparison of feature-fusion and resampling configurations on the test set under seed 42.

Configuration	P (%)	R (%)	mAP_50_ (%)	mAP_75_ (%)	mAP_50–95_ (%)	Params (M)	GFLOPs (1280 × 1280)	Inference FPS (1280 × 1280)
YOLO11n	82.72	78.66	87.11	81.63	75.15	2.583	25.3	252.37
YOLO11n + EMBSFPN	81.01	**81.72**	88.14	82.77	76.26	2.123	26.2	218.92
YOLO11n + FreqFusion	**85.35**	77.19	87.55	82.65	76.58	2.504	**24.7**	191.52
YOLO11n + HS-FPN	79.13	80.01	85.68	81.26	74.30	**2.072**	59.8	81.05
YOLO11n + DySample+	83.06	78.93	86.84	82.21	75.41	2.607	25.4	**274.57**
YEIS	82.51	81.13	**88.77**	**83.62**	**77.06**	2.149	26.9	206.44

All results were obtained on the test set under seed 42 using an input size of 1280 × 1280. GFLOPs and model-only inference FPS were evaluated at the same input size. Bold values indicate the best performance for each evaluation metric.

The detection results on typical samples and the heatmap responses further show that YEIS focuses more strongly on abnormal berry candidate regions inside grape clusters. Under challenging conditions such as color-similar backgrounds, dense berry distributions, branch and leaf occlusion, and strong specular reflection, the model can reduce some missed detections of abnormal berries. At the same time, the number of false positives increases to some extent. This indicates that, once the model becomes more sensitive to weak abnormal candidates, waxy bloom, specular highlights, normal berry skin textures, and adjacent berry boundaries may still be misclassified as abnormal berries. Overall, YEIS tends to improve the completeness of abnormal berry candidate detection, but false-positive suppression remains an aspect that requires further optimization.

Compared with YOLO11n, YEIS reduced the parameter count by 16.8% (2.583 M to 2.149 M), whereas throughput decreased by 18.2% (252.37 to 206.44 FPS). The advantage of YEIS therefore lies in higher Recall, mAP_75_, and mAP_50–95_ rather than inference speed.

### 4.2 Limitations and future work

Although YEIS reduces missed detections of abnormal berries, it also increases the number of false positives. In field images of Munage grapes, waxy bloom, specular highlights, normal berry skin textures, and adjacent berry edges can all produce local responses similar to those of abnormal berries. Future work may introduce confidence calibration, a false-positive suppression branch, or a two-stage verification strategy to re-filter weak abnormal candidate regions, thereby reducing the impact of false positives in practical applications.

Although YEIS reduced the parameter count, its on-device performance was not evaluated. Future work will investigate hardware-specific optimization and on-device testing on mobile inspection platforms or orchard robots.

In addition, the dataset used in this study was collected from a single year and a single orchard. The major and minor categories were defined as visual labels according to visible abnormal appearances in images, and were not intended for pathological diagnosis or causal determination of damage. In the future, data from multiple years, orchards, and grape cultivars can be introduced to improve the generalization ability of the model under different growth conditions, illumination environments, and abnormality types. In future work, we will continue collecting Munage grape images using ZED cameras and Mech-Eye NANO under different field conditions. These images will be combined with smartphone-acquired data to construct a multi-device dataset, enrich the diversity of real-world imaging conditions, reduce the potential bias associated with a single imaging device, and further evaluate and improve the model’s generalization across devices and field environments. For application extension, previous studies have achieved three-dimensional grape picking-point localization through key-structure detection, structural constraints, and three-dimensional geometric analysis (Zhou et al., 2025). Building on the abnormal berry detection results of this study, future work can further integrate RGB-D, multi-view, or multispectral information to explore collaborative mechanisms between abnormal berry recognition and robotic inspection, graded harvesting, or damage-minimizing harvesting decisions.

Unsupervised and weakly supervised learning provide alternatives when abnormal samples or bounding-box annotations are limited. Agricultural studies have used image-level labels and activation maps for weakly supervised pest classification (Bollis et al., 2022), while autoencoder-based methods reduce dependence on fully annotated abnormal samples (Strothmann et al., 2019; Miranda et al., 2022; de Villiers et al., 2023). This study used fully supervised detection because simultaneous class-specific localization of grape clusters and major and minor abnormal berries was required. Future work will examine weakly supervised localization and normal-only anomaly modeling for rare or previously unseen abnormalities.

## 5 Conclusion

This study focused on the simultaneous detection of Munage grape clusters and abnormal berries under color-similar backgrounds. A YOLO11n-based detection model, YEIS, was developed to address cross-scale targets, weak local abnormal cues, and interference from shallow detail responses. The main conclusions are as follows.

1. A detection dataset for Munage grape clusters and abnormal berries was constructed, containing 1,878 field images and three categories: GCLUSTER, major, and minor. Among them, major and minor were defined as visual labels according to visible abnormal appearances in the images. The visible regions of abnormal berries were used as detection boxes, and these labels were not intended for pathological diagnosis or causal analysis of damage. YEIS combines EMBSFPN for multi-scale feature construction with IB-FLED and SGRCM for local abnormal-cue enhancement and semantic-guided detail compensation.
2. YEIS achieved Precision, Recall, mAP_50_, mAP_75_, and mAP_50–95_ values of 83.21%, 79.97%, 88.34%, 83.27%, and 76.91%, respectively. Relative to YOLO11n, overall mAP_50–95_ increased by 1.71 percentage points; major-class mAP_50–95_ increased by 1.61 percentage points; and minor-class F1-score and mAP_50–95_ increased by 3.01 and 3.41 percentage points, respectively. The parameter count decreased by 16.8%, from 2.583 M to 2.149 M. The ablation and fixed-threshold error analyses were consistent with improved abnormal-berry detection, while the heatmaps provided qualitative support for more localized response patterns. YEIS provides a detection framework for vineyard monitoring and abnormal-berry recognition.

## Data Availability

The datasets generated for this study are available on request to the corresponding author.
